# P98 Prevalence of bacteria carrying ESBL genes on hospital surfaces in low- and middle-income countries

**DOI:** 10.1093/jacamr/dlaf230.105

**Published:** 2025-12-04

**Authors:** J Rzaska, A Cooke, R Farzana, B Hassan, C Achi, M Nieto-Rosado, J Oduwo, E Owinoh, R Zahra, K C Iregbu, A Mahboob, S Riaz, M H Hamid, A Muhammad, M Khan, E Rashid, N Medugu, O Oduyebo, I Nwafia, M Yahaya, A Aminu, L Wilson, S Langhorne, A Sindhu, A Rehman, A Shah, A Shoaib, R Tariq, R Temur, M Babar, A Sajid, R Razzaq, S Pervin, R Farzana, N Tasnim, T Akujobi, O Ogunsanya, M Ahmad, P Nwachukwu, I Orabueze, J Abubakar, S Chukwuma, T Walsh, K Sands, K Thomson

**Affiliations:** Ineos Oxford Institute for Antimicrobial Research (IOI), Department of Biology, University of Oxford, UK; Ineos Oxford Institute for Antimicrobial Research (IOI), Department of Biology, University of Oxford, UK; Ineos Oxford Institute for Antimicrobial Research (IOI), Department of Biology, University of Oxford, UK; Department of Medical Microbiology, Cardiff University, UK; Ineos Oxford Institute for Antimicrobial Research (IOI), Department of Biology, University of Oxford, UK; Ineos Oxford Institute for Antimicrobial Research (IOI), Department of Biology, University of Oxford, UK; Ineos Oxford Institute for Antimicrobial Research (IOI), Department of Biology, University of Oxford, UK; Ineos Oxford Institute for Antimicrobial Research (IOI), Department of Biology, University of Oxford, UK; Department of Microbiology, Quaid-i-Azam University, Islamabad, Pakistan; National Hospital Abuja, Department of Medical Microbiology, Abuja, Nigeria; Bacha Khan Medical Complex, Swabi, Pakistan; Pakistan Institute of Medical Sciences, Islamabad, Pakistan; Mayo Hospital, Lahore, Pakistan; Lady Reading Hospital, Peshawar, Pakistan; National Institute of Child Health (NICH) and Jinnah Postgraduate Medical Centre (JPMC), Karachi, Pakistan; Department of Microbiology, Quaid-i-Azam University, Islamabad, Pakistan; National Hospital Abuja, Department of Medical Microbiology, Abuja, Nigeria; Massey Street Children Hospital, Lagos, Nigeria; University of Nigeria Teaching Hospital, Enugu, Nigeria; Usmanu Danfodiyo University Teaching Hospital/Sokoto State Specialist Hospital, Sokoto, Nigeria; Aminu Kano Teaching Hospital/Bayero University Kano, Nigeria; School of Energy, Geoscience, Infrastructure and Society, Heriot-Watt University, Edinburgh, UK; Ineos Oxford Institute for Antimicrobial Research (IOI), Department of Biology, University of Oxford, UK; Ineos Oxford Institute for Antimicrobial Research (IOI), Department of Biology, University of Oxford, UK; Bacha Khan Medical Complex, Swabi, Pakistan; Bacha Khan Medical Complex, Swabi, Pakistan; Pakistan Institute of Medical Sciences, Islamabad, Pakistan; Pakistan Institute of Medical Sciences, Islamabad, Pakistan; Mayo Hospital, Lahore, Pakistan; Mayo Hospital, Lahore, Pakistan; Lady Willingdon Hospital, Lahore, Pakistan; Lady Willingdon Hospital, Lahore, Pakistan; Khulna Medical College Hospital, Khulna, Bangladesh; Dhaka Medical College Hospital, Dhaka, Bangladesh; Mymensingh Medical College Hospital, Mymensingh, Bangladesh; National Hospital Abuja, Department of Medical Microbiology, Abuja, Nigeria; Massey Street Children Hospital, Lagos, Nigeria; Barnards-Balance Laboratory, Murtala Muhammad Specialist Hospital, Kano, Nigeria; University of Nigeria Teaching Hospital, Enugu, Nigeria; Parklane Hospital, Enugu, Nigeria; Usmanu Danfodiyo University Teaching Hospital/Sokoto State Specialist Hospital, Sokoto, Nigeria; Parklane Hospital, Enugu, Nigeria; Ineos Oxford Institute for Antimicrobial Research (IOI), Department of Biology, University of Oxford, UK; Ineos Oxford Institute for Antimicrobial Research (IOI), Department of Biology, University of Oxford, UK; Ineos Oxford Institute for Antimicrobial Research (IOI), Department of Biology, University of Oxford, UK

## Abstract

**Background:**

The hospital environment can be a hotspot for the emergence of antimicrobial resistance (AMR), with high antibiotic usage and varied cleaning practices acting as a reservoir for AMR bacteria that can persist on surfaces such as bedside tables, door handles or medical equipment. In BARNARDS-II (Burden of Antimicrobial Resistance in Neonates in Developing Societies), incidence, AMR and antibiotic usage in neonatal sepsis are evaluated. Within the study, we also investigate whether hospital surfaces can contribute to the spread of MDR bacteria.

**Objectives:**

To investigate potential sources of infection and determine the prevalence of ESBLs among bacteria colonizing hospital surfaces in maternity, delivery and neonatal wards across the BARNARDS network.

**Methods:**

Charcoal swabs were used to collect samples from low- and high-touch surfaces across neonatal, delivery and maternity wards for a period of three months in 13 hospitals across Bangladesh, Pakistan and Nigeria and shipped to Oxford under UN3373 regulations. Swabs were plated on non-selective and selective (cefotaxime and vancomycin) Chromatic Detection agar. Growth from antibiotic-supplemented media was recorded and multiplex PCRs were performed to assess the presence of ESBL resistance genes (*bla*_CT-XM15_, *bla*_OXA-1_, *bla*_SHV-1_ and *bla*_TEM-1_). Bacterial communities PCR positive for at least one target ARG were purified and separated to test morphologically distinct colonies and retested. Positive isolates were identified via MALDI-TOF MS.

**Results:**

Out of 1854 swabs processed to date, 1636 produced microbial growth on non-selective media and 1271 samples produced growth on Chromatic Detection plates supplemented with vancomycin and cefotaxime. PCR testing for key ESBL genes revealed that 353 samples were positive for at least one gene, with a total of *n*=585 isolates recovered. The gene most commonly found was *bla*_CT-XM15_ (*n*=455), followed by *bla*_TEM-1_ (*n*=362), *bla*_OXA-1_ (*n*=272) and *bla*_SHV-1_ (*n*=120). Out of all the isolates recovered, 33% isolates carried one gene (*n*=193), 32.8% carried two (*n*=192), 28.4% carried three (*n*=166) and 5.8% carried all four (*n*=34). The most common species carrying ESBLs were *Klebsiella pneumoniae* (*n*=117, 20%), followed by *Enterobacter hormaechei* (*n*=87, 14.87%) and *Klebsiella oxytoca* (*n*=66, 11.28%). The majority of the *K. pneumoniae* isolates (*n*=105, 89.7%) carried more than one ESBL, with *bla*_CT-XM15_ being the most prevalent ARG across the species (*n*=105, 89.7%). ESBL-carrying bacteria were found on a variety of surfaces, including medication trolleys, sinks and bedside tables across the wards.

**Conclusions:**

Our findings revealed a widespread microbial contamination across a range of surfaces in all ward types tested, with a substantial proportion of these being clinically relevant bacteria carrying antibiotic resistance genes. The prevalence of ESBLs in these strains indicates that hospital surfaces serve as a significant reservoir of MDR bacteria. This highlights the role of the hospital environment as a reservoir of pathogenic bacteria and the contribution to the burden of AMR in LMICs. Future work will include further environmental sampling and comparison of environmental and blood culture isolates from the same locations to investigate transmission networks.

